# Molecular signaling along the anterior–posterior axis of early palate development

**DOI:** 10.3389/fphys.2012.00488

**Published:** 2013-01-07

**Authors:** Tara M. Smith, Scott Lozanoff, Paul P. Iyyanar, Adil J. Nazarali

**Affiliations:** ^1^Laboratory of Molecular Cell Biology, College of Pharmacy and Nutrition, University of SaskatchewanSaskatoon, SK, Canada; ^2^Department of Anatomy, Biochemistry and Physiology, University of Hawaii School of MedicineHonolulu, HI, USA

**Keywords:** anterior–posterior axis, secondary palate, development, signaling, migration, growth factors

## Abstract

Cleft palate is a common congenital birth defect in humans. In mammals, the palatal tissue can be distinguished into anterior bony hard palate and posterior muscular soft palate that have specialized functions in occlusion, speech or swallowing. Regulation of palate development appears to be the result of distinct signaling and genetic networks in the anterior and posterior regions of the palate. Development and maintenance of expression of these region-specific genes is crucial for normal palate development. Numerous transcription factors and signaling pathways are now recognized as either anterior- (e.g., *Msx1, Bmp4, Bmp2, Shh, Spry2, Fgf10, Fgf7, and Shox2*) or posterior-specific (e.g., *Meox2, Tbx22, and Barx1*). Localized expression and function clearly highlight the importance of regional patterning and differentiation within the palate at the molecular level. Here, we review how these molecular pathways and networks regulate the anterior–posterior patterning and development of secondary palate. We hypothesize that the anterior palate acts as a signaling center in setting up development of the secondary palate.

## Introduction

Cleft palate is one of the most common congenital birth defects in humans, occurring with a frequency of 1:700 to 1:1000 live births (Gorlin et al., 2001). A cleft secondary palate can occur as an isolated birth defect (non-syndromic), in conjunction with a cleft lip, or as a part of another syndrome. Both genetic and environmental factors play roles in the development of cleft palate (Dixon et al., 2011).

During mammalian embryogenesis, the development of the secondary palate is regulated by a number of complex networks of growth factors and transcription factors. These molecular networks and pathways work together to tightly regulate critical cellular processes in the palate including cell proliferation, apoptosis, migration, and epithelial-mesenchymal transdifferentiation. The secondary palate originates from first branchial arch neural-crest derived mesenchymal cells covered by a multi-layer sheet of cells derived from the facial ectoderm (Noden, 1983). In the mouse, bilateral palate shelves first develop as outgrowths from the maxillary processes at embryonic day 11.5 (E11.5). The shelves then grow vertically down either side of the tongue until E14.0 (Ferguson, 1988), after which the shelves undergo a rapid elevation to become horizontally oriented toward one another above the tongue. Growth of the stomodeum as well as jaw joint activity and neuromuscular function make it possible for the embryo to have mouth-opening reflexes. These movements allow the tongue to flatten and depress, and the downward positioned palate shelves to reorient (Humphrey, 1969; Diewert, 1980). A number of changes occur within the palate shelves to facilitate the rapid movement of the shelves from a vertical to a horizontal position starting at the anterior end and proceeding posteriorly, however, a clear understanding of how elevation occurs has yet to be achieved. Ultimately, the elevated palatal shelves then grow toward one another until the medial edge epithelium from each shelf contacts to form the midline epithelial seam (MES) at E14.5. In addition to growth of the palate shelves, a change in the relative dimensions of the head (vertical dimensions of the head increase while the lateral maxillary width remains constant) allows the palate shelves to contact one another at the midline (Diewert, 1978, 1983). Epithelial cells from opposing palate shelves adhere to one another through glycoproteins on their surface (Greene and Kochhar, 1974; Pratt and Hassell, 1975; Souchon, 1975; Greene and Pratt, 1977) as well as through desmosomes (De Angelis and Nalbandian, 1968; Morgan and Pratt, 1977). Contact and subsequent fusion begins in the anterior mid-palate regions and proceeds in both the anterior and posterior directions like a zipper (Morgan and Pratt, 1977; Ferguson, 1988). The MES then undergoes a rapid degradation to form a secondary palate with complete mesenchymal confluence (Ferguson, 1988; Berkovitz et al., 2009). Numerous mechanisms for the degradation of the MES have been proposed, including epithelial apoptosis (Pourtois, 1966; Saunders, 1966; Farbman, 1968; Shuler, 1995; Martínez-Álvarez et al., 2000a; Xu et al., 2006), migration (Carette and Ferguson, 1992; Shuler et al., 1992; Martínez-Álvarez et al., 2000a), and epithelial-mesenchymal transformation (Fitchett and Hay, 1989; Griffith and Hay, 1992; Shuler et al., 1992; Kaartinen et al., 1995; Proetzel et al., 1995; Sun et al., 1998; Cui et al., 2005). Epithelial-mesenchymal transformation has been ruled out based on fate-maps (Vaziri Sani et al., 2005), but this theory is still unsettled. Mesenchymal confluence signals the end of palatogenesis at E15.5 (Ferguson, 1988). Finally, the anterior secondary palate fuses to the primary palate and the dorsal portions of the secondary palate fuse with the nasal septum marking the completion of proper palatal development. Distinct pathways/networks regulate development at each stage of palatogenesis, with defects at any stage capable of resulting in cleft palate. In addition to problems with development of the palate proper, defects in the development of other craniofacial elements including the tongue and mandible can result in a cleft palate (Ferguson, 1987).

**Figure 1:**
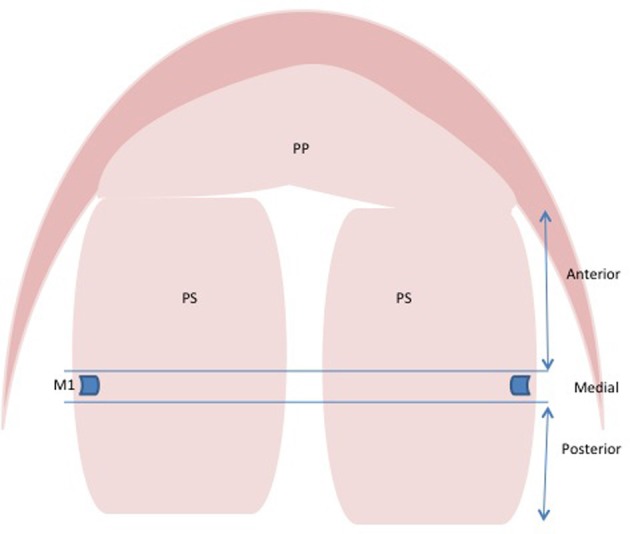
**Schematic representation for defining anterior, medial, and posterior regions of palatal shelves.** Tissue anterior or posterior to first molar tooth is considered anterior or posterior palate, respectively. The palatal tissue in the region of first molar tooth bud is considered medial. Abbreviations: PP, primary palate; PS, palatal shelves; M1, first molar tooth bud.

Analysis of the literature on regionally expressed genes can be difficult since a standardized method of determining the anterior, medial, and posterior regions of the palate is not in place. Many authors fail to indicate how they define the region of the palate that they are examining making comparison difficult between articles. It is important for the field to adopt a standard convention for defining the anterior, medial, and posterior palate to ensure that these comparisons can be made. In the past, the anterior and posterior have been described in a number of ways. We propose that the convention can be followed such that the tissue anterior or posterior to the first molar tooth bud be considered the anterior or posterior palate, respectively. The medial palate would be considered palate tissue in the plane of the molar tooth bud (Figure 1). The rationale is that the first formed palatal rugae (R1) demarcates the expression boundary of anterior (e.g., *Msx1, Shox2, and Fgf10*) and posterior (e.g., *Meox2, Tbx22*) specific genes (Zhang et al., 2002; Yu et al., 2005; Li and Ding, 2007; Pantalacci et al., 2008; Welsh and O'Brien, 2009; Bush and Jiang, 2012). The first molar tooth bud lies immediately anterior to the R1, which forms the posterior boundary for anterior *Fgf10* expression (Welsh et al., 2007). On the structural basis, the anterior two-thirds of the palate is the future hard palate. During rostral extension of the anterior palate from E11.5 to E14.5, the spatial relationship between R1 and the developing molar tooth bud remains unchanged (Welsh et al., 2007; Welsh and O'Brien, 2009), making the molar tooth bud an ideal convention to delineate the two structurally distinct regions of palate.

This review will provide an in depth look at the molecular processes involved in regulating the patterning and early development of the secondary palate. Genes known to be involved in the fusion of the palate processes will not be discussed in detail; see Nawshad (2008) for a comprehensive review. The major focus here will be to summarize both current information and developing new connections between the factors and genes involved in specifying and maintaining the A–P axis. We hypothesize that the anterior palate acts as a signaling center for secondary palate patterning and development.

## Anterior-specific gene expression

**Figure 2:**
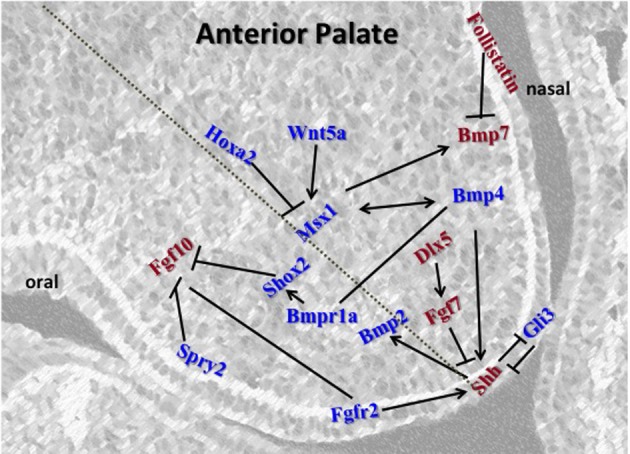
**Schematic representation of the key regulators in the anterior palate.** *Msx1* and *Bmp4* function in an autoregulatory loop mechanism in the mesenchyme. *Bmp4* induces *Shh* expression in the epithelium which signals backs to the mesenchyme to positively regulate *Bmp2* to enhance cell proliferation in the mesenchyme. *Msx1* expression is controlled by Hoxa2 in early palatal development. *Fgfs* and their receptors are regulated by *Spry*2 for proper balance of proliferation and prevention of premature apoptosis in the epithelium. *Fgf10* induces *Shh* whereas *Fgf*7 acts as an antagonist. *Msx1* also maintains proliferation by inducing *Bmp*7 in the mesenchyme along the nasal epithelium. Genes represented in red are restricted to either oral or nasal side of the palate, whereas those represented in blue are present across the shelf.

A large number of anterior-specific genes specifically expressed and active within the anterior palate (Figure 2) compared to the posterior palate highlights the importance of the anterior region during secondary palate development (Zhang et al., 2002; Rice et al., 2004; Alappat et al., 2005; Yu et al., 2005; Levi et al., 2006; Lee et al., 2007, 2008; Welsh et al., 2007; He et al., 2008; Liu et al., 2008).

## Msx1 network

The first network described to play an anterior-specific role in the developing palate involves the homeobox transcription factor *Msx1*. Mutations in the human *MSX1* gene have been linked to isolated non-syndromic cleft palate (Vastardis et al., 1996; Lidral et al., 1998; Van den Boogaard et al., 2000; Suzuki et al., 2004; Tongkobpetch et al., 2006; Otero et al., 2007). *Msx1*-deficient mice display neonatal lethality due to a wide open cleft secondary palate (Satokata and Maas, 1994; Houzelstein et al., 1997). Expression of *Msx*1 is localized exclusively to the anterior mesenchyme during the early stages of palate development from E12.5 to E13.5 (Zhang et al., 2002; Alappat et al., 2003) and functions through regulating the expression of *Bmp4, Shh*, and *Bmp2* at E12.5–E13.5 in the anterior palate (Zhang et al., 2002). *Msx1* appears to regulate *Bmp4* expression in the anterior mesenchyme, which subsequently signals to the epithelium and regulates *Shh* expression; from the epithelium *Shh* then signals back to the mesenchyme and regulates *Bmp2* expression (Zhang et al., 2002). In addition to this linear network, *Bmp4* is involved in a reciprocal regulatory cycle controlling the expression of *Msx1*. The main function of *Msx1* and its subsequent network appears to be regulation of cell proliferation within the anterior mesenchyme (Zhang et al., 2002). It has been demonstrated that although exogenous BMP is capable of inducing *Msx1* expression and increasing cell proliferation in the anterior palate it has no effect on the posterior palate (Zhang et al., 2002; Hilliard et al., 2005). Since first reported, numerous studies have investigated the expression and function of the genes in this network. All studies performed to date (see below) confirm that this network is important in anterior mesenchyme proliferation; however, the regulation of each of these genes is far more complex than suggested originally.

The regulation of *Msx*1 expression has been linked to many other factors in the palate. *Msx1* was shown to be downstream of the *Foxe1* gene with *Foxe1* null mice having very low expression of palatal *Msx1* and *Tgf-β3* at E14 (Venza et al., 2011). Loss of the Fgf antagonist *Spry2* in the piebald deletion animal model (discussed in more details in the section “Fgf Signaling Pathway”) results in increased *Msx1* expression as well as a posterior expansion of its expression border at E13.5 and E14.5; this increased expression leads to an increase in proliferation within the palate (Welsh et al., 2007). *Msx1* is Fgf-responsive in other regions of craniofacial development (Bei and Maas, 1998; Alappat et al., 2003), although *Fgf10* null palates do not exhibit altered *Msx1* expression (Alappat et al., 2005). These data suggest other Fgfs may be acting in the palate to regulate *Msx1* expression (other possible explanations are discussed in subsequent sections below). *Fgf9* may play an active role in palate development (Colvin et al., 1999, 2001) and loss of *Spry*2 may relieve the antagonism of *Fgf9* resulting in the observed upregulated and expanded *Msx1* expression (Welsh et al., 2007). Recently, *Fgf9* was shown to regulate cell proliferation in palatal mesenchyme via *Pitx2*-dependent induction of cyclin D1 and cyclin D3 in the *Tgfbr2^fl/fl^*; *Wnt1-Cre* mice (Iwata et al., 2012a), however, expression of *Msx1* was not examined in this study. *Fgf7* is expressed within the palate mesenchyme (Rice et al., 2004) and may also be involved in regulating *Msx1* expression and affected by loss of *Spry2*, although this has not been investigated.

*Hoxa2*, another homeobox gene, has recently been shown to regulate palatal *Msx1* expression (Smith et al., 2009). *Hoxa*2 null mice exhibit an 81% penetrance of cleft palate (Gendron-Maguire et al., 1993; Rijli et al., 1993; Barrow and Capecchi, 1999), which appears to result from increased cell proliferation where expression levels of both *Msx1* and its known down-stream target *Bmp4* are up-regulated during the early stages of palate development (Smith et al., 2009). Genetic studies in humans have also linked mutations in the *HOXA2* gene with a cleft secondary palate (Alasti et al., 2008). *Hoxa*2 acts upstream of *Msx1* in the second branchial arch neural crest cells (Santagati et al., 2005). This new gene target provides additional insight, as *Hoxa2* is known to be absent from the migrating first branchial arch from which the palate shelves arise (Prince and Lumsden, 1994). Clearly expression in the branchial arches prior to overt palate growth is not a prerequisite of genes that are important in regulating palatogenesis. Whether *Hoxa*2 and Fgfs represent distinct regulatory network of *Msx1* or are part of the same regulatory network remains to be determined. Strict regulation of *Msx1* expression in the palate is probably due to its importance in regulating proliferation in the anterior palate.

The transcriptional activity of *Msx1* can also be altered by other proteins in the palate. *Msx1* undergoes post-translation modification by sumoylation *in vivo* in a region of the protein that is responsible for regulating *Msx1* interactions with other proteins (Gupta and Bei, 2006). Thus, sumoylation of *Msx1* may help facilitate its ability to interact with other transcription factors and therefore control its ability to regulate the expression of other genes. Haploinsufficiency of the SUMO1 gene has been reported to lead to cleft palate through altering the sumoylation status of various proteins (*Eya1, Pax9*, and *Msx1*) in the palate (Alkuraya et al., 2006). However, it has also been suggested that SUMO1 expression is not necessary for normal mouse development (Zhang et al., 2008). Debates also exist on whether polymorphisms of the SUMO1 gene in humans are linked to cleft palate (Song et al., 2008; Almeida de Assis et al., 2011). What role SUMO1 plays in palate development is therefore unclear at this time.

In addition to regulating *Bmp4* and *Bmp2, Msx1* regulates the expression of Bmp7 and its antagonist Follistatin (Levi et al., 2006). Loss of *Msx1* leads to a decrease in the anterior palatal expression of *Bmp7* but an increase in its expression in the posterior palate (Levi et al., 2006). The Bmp antagonist Follistatin is expressed throughout the palatal epithelium; in the anterior palate it is primarily expressed in a restricted dorsal domain that does not overlap the regions of *Bmp4* and *Bmp2* expression (Levi et al., 2006). *Msx1* null mice also exhibit a decrease in the level of anterior palatal Follistatin expression (Levi et al., 2006). Together, these data highlight the important role of *Msx*1 in the regulation of the Bmp family and their antagonists in the palate, and provide another mechanism by which it may regulate the level of proliferation in the anterior palate.

*Dlx5* is expressed in the anterior mesenchyme of the palate and mutations in the *Dlx5* gene result in a cleft secondary palate (Levi et al., 2006; Han et al., 2009). Furthermore, loss of the transcription factor MEF2C consequently leads to loss of *Dlx*5 expression in the branchial arches resulting in a cleft palate (Verzi et al., 2007). Although *Dlx5* and *Msx1* share similar expression domains it is unlikely that they are involved in regulating each others expression as *Msx1* expression is not altered in *Dlx5* null palates and vice versa (Han et al., 2009). *Dlx5/Msx1* double knockouts show a rescue of the *Msx1* null cleft palate phenotype (Levi et al., 2006; Han et al., 2009). Loss of *Dlx5* in *Msx*1 null embryos alters the expression of *Shh, Bmp7*, and *Follistatin* in the palate. Bmp7 expression in these double knockouts is increased throughout the palate, while expression of Follistatin is decreased (Levi et al., 2006; Han et al., 2009). *Shh* expression is decreased in *Msx1* null palates but its domain is expanded in the double knockouts suggesting that both *Msx1* and *Dlx5* are involved in determining the area of *Shh* expression (Han et al., 2009). *Dlx5* and *Fgf7* share the same expression region in the anterior palate mesenchyme on the nasal side. *Fgf7* region of expression is limited in *Dlx5* null mutants as well as in the *Msx1/Dlx5* double knockouts (Han et al., 2009). These data point toward a system where *Dlx5* regulates the expression of *Fgf7*, which in turn represses *Shh.* It has also been demonstrated that a feedback loop and cross talk exists between *Bmp7* and *Shh*, which plays a role in refining the expression domain of both genes (Han et al., 2009). Therefore, in the *Msx1/Dlx5* double knockouts the limited *Bmp7* expression allows an increase in *Shh* expression, which likely leads to the observed increase in cell proliferation and rescues the *Msx1*-induced cleft palate.

## Bone morphogenic protein signaling pathways

*Bmp4* is known to be downstream of *Msx*1 in the palate (Zhang et al., 2002). However, similar to *Msx1*, many alternative regulatory pathways for *Bmp4* have been described in recent years. The transcription factor *Tbx3* shows an overlapping expression pattern with *Bmp4* in the developing anterior palate mesenchyme (Lee et al., 2007). These two genes regulate each other's expression in the palate whereby *Tbx3* inhibits the expression of *Bmp4* while *Bmp4* induces *Tbx3* expression (Lee et al., 2007). As expected and based on the previously reported role of *Bmp4* in the palate, this regulatory loop acts by regulating the levels of cell proliferation in the anterior palatal mesenchyme (Lee et al., 2007). In the limb, *Tbx3* expression is dependent on *Bmp4* (Tümpel et al., 2002) and plays an important role in maintaining normal proliferation in the region (Davenport et al., 2003). *Tbx3* null embryos however, do not exhibit a cleft palate (Davenport et al., 2003) and therefore the ability of *Tbx3* to regulate *Bmp4* expression and subsequently proliferation may be redundant with another regulatory mechanism in the palate.

At the onset of palate development, the transcription factor *Tp63* regulates the expression of *Bmp4* in the anterior palate. Loss of the *Tp63* gene leads to cleft palate through altering the expression of a variety of genes (including *Bmp4*) in the maxillary processes from which the palatal shelves emanate. This altered gene expression results in defects of the A–P axis as well as the onset of palate development (Thomason et al., 2008). These observations indicate regulation of gene expression during and prior to the overt growth of the palate shelves can influence palate development and patterning.

*Bmp4* acts upstream of *Shh* and *Bmp2* within the palate (Zhang et al., 2002). New studies detail the importance of the *Wnt5a* signaling molecule in regulating the A–P axis in the palate including the expression of *Bmp4* (He et al., 2008). In the absence of *Wnt5a* signaling, *Bmp4* expression is down-regulated in the anterior palate at E13.5, while being ectopically up-regulated in the posterior palate (He et al., 2008). As predicted, *Shh* expression in the anterior palate and posterior palate correspondingly decreases and increases, respectively. Surprisingly, *Bmp2* expression was unaltered in the *Wnt5a* null mutants (He et al., 2008), implying *Bmp2* expression in the palate is regulated by an additional mechanism. Despite a decrease in *Bmp4* and *Shh* expression, proliferation was increased in the anterior mesenchyme, which is contrary to what would normally be expected (He et al., 2008).

Noggin is a polypeptide that binds to members of the Bmp family preventing them from signaling. Noggin null mice show that without Noggin's repression of Bmp signaling, palate development does not proceed normally, with fusion between the palate and mandible ultimately leading to a cleft palate phenotype. Although Noggin null mice did not have changes in the expression of *Msx1, Bmp4*, or *Shh* they did have reduced *Shox2* and *Bmp2* expression in the anterior palate and an ectopic extension of *Bmp2* expression into the posterior region of the palate. In addition, decreased proliferation rates were seen exclusively in the anterior mesenchyme of Noggin null palate which suggests that loss of *Bmp2* in the anterior palate effects proliferation and supports the theory that posterior cells are not receptive to ectopic Bmp expression (Hilliard et al., 2005; He et al., 2010).

The Bmp family plays an important role in maintaining the A–P axis of the palate shelves as well as regulating proliferation (Nie, 2005). *Bmp* ligands regulate down-stream gene expression and cell processes through activation of cellular receptors. *Bmpr1a* and *Bmpr1b* are expressed in an overlapping pattern in the anterior palate. The Bmp receptor Bmpr1a is essential in the regulation of proliferation and patterning in the palate; a total loss of the *Bmpr1a* gene in all craniofacial cells leads to decreased proliferation as well as an anterior shift in the expression patterns of the posterior-specific genes *Pax9* and *Barx*1 (Liu et al., 2005). Conditional loss of *Bmpr1a* in the neural crest and derivatives (*Wnt1-Cre*; *Bmpr1a*^f/−^ mice) leads to an anterior clefting of the secondary palate resulting from decreased mesenchymal proliferation (Li et al., 2011). The significantly reduced expression of *Msx1, Bmp4, Pax9*, and *Shox2* may be responsible for the defective cell proliferation. These results indicate that although Bmpr1b has a common expression pattern it is not able to compensate for the loss of epithelial Bmpr1a expression (Li et al., 2011). Interestingly when Bmpr1a is deleted from all craniofacial tissue the expression of *Msx1* is unaltered (Liu et al., 2005). Together these data show that the role and number of Bmp receptors in the palate is complex and yet to be fully understood. This could also imply a novel role for *Bmp4* and potentially other Bmps acting through the Bmpr1a receptor in regulation of the spatial expression of posterior-specific genes.

## Sonic hedgehog signaling

Sonic hedgehog *(Shh)* is expressed in the epithelium throughout palatogenesis (Paiva et al., 2010) and proper regulation of the *Shh* signal is crucial for normal palate development to occur. Expression is restricted to a striped pattern that corresponds to the rugae (Rice et al., 2006). Rugae develop through the thickening of the epithelium and condensation of the underlying mesenchyme. These rugae are suggested to act as centers that coordinate patterning within the palate implying an important role for *Shh* in the patterning of the developing palate (Rice et al., 2004; Lin et al., 2011). The epithelial cells expressing *Shh* are not actively proliferating, whereas the mesenchymal cells underlying these regions are more highly proliferative than mesenchymal cells in other areas of the palate (Han et al., 2009). Loss of rugae and rugae-specific morphogens has been suggested to hamper the molecular guidance necessary to regulate the growth of the palate. For example, loss of Wnt signaling in the palate epithelium blocks the formation of the rugae and altered *Shh* expression which in turn results in abnormal extension along the A–P axis and a unique anterior only cleft palate phenotype (Lin et al., 2011). *Shh* is also a down-stream target of the *Msx1* network that regulates cell proliferation in the anterior palate (Zhang et al., 2002). Loss of the *Spry2* gene also leads to a disorganization in the expression pattern of *Shh*, which ultimately leads to deformities in the rugae in the palate of these knockout animals (Welsh et al., 2007). Double null mutants of Fgf intracellular antagonists *Spry2*^−/−^ act as Fgf gain-of-function mutant with highly disorganized palatal rugae. Similar rugae disorganization was also observed in the conditional deletion of *Shh* (*K14*-*Cre*; *Shh*^*fl/fl*^ *mice*) (Economou et al., 2012). Their analyses suggests that Fgf acts as an activating factor and *Shh* acts like *Spry*, functioning as an inhibitor of Fgf signaling and of rugae development.

Gli3, a protein expressed in the epithelium and mesenchyme along the entire A–P axis of the palate, is capable of acting as both an activator and repressor of *Shh* signaling (Huang et al., 2008). In the absence of the *Shh* signal, the Gli3 protein is processed by protein kinase A allowing it to enter the nucleus and repress the expression of *Shh* target genes. Presence of the *Shh* signal prevents the processing of the Gli3 protein, and therefore prevents Gli3 from repressing the expression of the *Shh* target genes (Wang et al., 2000; Litingtung et al., 2002). In the limb, an antagonistic relationship between *Shh* and Gli3 is crucial in setting up the A–P axis. Gli3 is expressed in the anterior region of the developing limb, where it represses the expression of *Shh*. dHAND is a posterior-specific protein in the limb that is also repressed by Gli3 but is a known activator of *Shh* expression. Together, this pathway sets up an A–P axis in the limb that ensures proper development (Niswander, 2003). This important interaction between Gli3 and *Shh* in the limb in combination with the expression of both genes in the palate suggests a role for Gli3 in the palate. Not surprisingly, *Gli3* null mice display a cleft secondary palate; however, the cleft palate phenotype was not due to changes within the palate itself, but rather due to defective growth of the tongue (Huang et al., 2008). These results demonstrate that regional differences and signaling pathways are not conserved between areas of the developing embryos. Hence, simply lining up the expression of all of the players in a pathway within the palate does not necessarily imply they function by a similar mechanism as described for other areas of the developing embryo.

## Fgf signaling pathway

Mutations in numerous members of the Fgf family have been linked to cleft palate in the human population (Riley et al., 2007). The best understood Fgf-dependent pathway in the palate involves *Fgf10* and its receptor *Fgf2rb*. *Fgf10* null mice exhibit a wide open cleft palate that is due to abnormal palate shelf morphology and size, preventing the shelves from contacting at the MES (Rice et al., 2004; Alappat et al., 2005). In addition, ectopic fusion of the palate shelves to the oral epithelium is observed in some animals, preventing normal shelf elevation (Alappat et al., 2005). Similar to *Msx*1 expression, *Fgf10* is expressed primarily in the anterior palate mesenchyme at the early stages (E12–E13) of palatogenesis (Rice et al., 2004). The *Fgf10* ligand acts through the receptor *Fgfr2b*, which also shows an anterior-specific expression pattern in areas of epithelium adjacent to mesenchyme expressing *Fgf10* (Rice et al., 2004). *Shh* expression is down-regulated in the epithelium of both *Fgf10* and *Fgfr2b* null embryos, leading directly to a severe reduction in epithelial cell proliferation and a consequently thin epithelial layer. Mesenchymal cell proliferation also significantly decreases due to a lack of reciprocal *Shh* signaling through its receptor *Ptc*1 (Rice et al., 2004). As discussed above, *Msx*1 expression is not altered in *Fgf10* null mutants (Alappat et al., 2005), nor is *Bmp4* expression. However, the Bmp antagonist Sostdc1 does have reduced expression levels in *Fgf10* null palates (Welsh and O'Brien, 2009). Therefore the altered *Shh* expression in the *Fgf10* or *Fgfr2b* null mice may be due to the decreased antagonism on Bmp signaling or directly due to loss of Fgf signaling and not through alterations in the *Msx*1 network. Conditionally knocking out all *Fgfr*2 isoforms exclusively in the epithelium also lead to a cleft palate. Once again *Shh* expression is disordered and there is a lack of clearly defined rugae during the time palatogenesis normally occurs. In this instance however, cell proliferation is only decreased within the epithelium, suggesting that *Fgfr*2 receptors must exist in mesenchymal cells, and be responsible for regulating cell proliferation in these cells (Hosokawa et al., 2009). Loss of *Fgf*10 signaling alters cellular processes including apoptosis, suggesting it plays a role in cell survival. *Fgf10* null mice exhibit premature and ectopic fusion indicating that *Fgf10* also has a role in ensuring proper fusion (Rice et al., 2004).

While *Fgf10* regulates cell proliferation within the anterior palate, ectopic exposure to *Fgf10* does not bring about a noticeable effect on the level of proliferation in the posterior palate (Yu et al., 2005), implying the down-stream effectors of *Fgf10* expression must not be present within the posterior region of the palate. This provides further evidence the anterior and posterior regions of the palate are distinct cell populations with very different regulatory mechanisms for the same cellular processes.

In addition to regulating proliferation, apoptosis, and fusion, *Fgf10* can also induce cell migration within the anterior palate. *Fgf10* expression is not only localized to the anterior mesenchyme but is also higher in the oral region of the anterior mesenchyme (Rice et al., 2004). *Fgf10* acts as a chemoattractant and induces the migration of anterior mesenchyme cells from the nasal to the oral side of the palate (He et al., 2008). The loss of *Fgf10* causes palate shelves to assume an abnormal shape (Alappat et al., 2005), which could in part be explained by the loss of oral cell migration.

The Fgf receptor *Fgfr1b* has also been described as having an anterior and nasal specific expression pattern within the developing palate (Lee et al., 2008). As with other members of the Fgf family, its expression is linked to the regulation of proliferation within the anterior palate. Expression of *Fgfr1b* is negatively regulated by the *Wnt11* signaling molecule. In return, *Fgfr1b* negatively regulates the expression of *Wnt11* (Lee et al., 2008). At early stages (E13.5) of palate development, the balance is tilted toward *Fgfr1b* allowing the palate to undergo cell proliferation. However, as palate development proceeds (E14), the expression balance is shifted away from *Fgfr1b*. At this stage proliferation must temporarily halt in order for the individual palate shelves to fuse and form a complete palate (Lee et al., 2008). Thus, although the most obvious role for the Fgf family is in regulating the level of proliferation within the palate, the Fgf family also plays a role in regulating events such as fusion by maintaining minimal expression of certain genes until the appropriate time.

As discussed above, one specific animal model showed that loss of the Fgf antagonist *Spry2* leads to a cleft palate due to alterations in the level of cell proliferation within the palate as well as the expression profiles of numerous genes including *Msx1* (Welsh et al., 2007; Matsumura et al., 2011). *Spry2* is expressed in the epithelium and mesenchyme at consistent levels throughout palatogenesis (Matsumura et al., 2011). The animal model discussed above has a piebald deletion, which is a collection of overlapping Mb-scale chromosomal deficiencies which includes the *Spry2* gene (Welsh et al., 2007), while another is a single specific knockout of the *Spry2* gene (Matsumura et al., 2011). Earlier reports from the group that developed the animal model lacking exclusively *Spry2* indicated that animals were not shown to have a cleft palate (Shim et al., 2005; Taketomi et al., 2005), however more recent reports have shown a prevalence for cleft palate (Matsumura et al., 2011). The piebald deletion led to a high incidence of cleft palate while the targeted deletion of *Spry2* only displayed the cleft palate phenotype in approximately 20% of animals. The differences in incidence rates are likely due to other defects resulting from the Mb-scale of the piebald deletion. Both mutants showed that a loss of *Spry2* expression leads to an increase in the level of cell proliferation in the palate (Welsh et al., 2007; Matsumura et al., 2011) which could be expected since its absence relieves inhibition on Fgf signaling which has been reported to control proliferation rates (Rice et al., 2004; Alappat et al., 2005). Also seen in both mutant animal models was altered *Msx1* expression. The true knockout model showed an increase in the level of *Msx1* expression, although region specific expression was not investigated (Matsumura et al., 2011). The piebald mutation initiates a posterior expansion of *Msx*1 coinciding with a loss of the anterior expansion of the posterior-specific transcription factor *Tbx22*. While *Tbx22* expression fails to reach its normal anterior expression boundary, *Etv5* and *Barx1*, which are primarily expressed in the posterior palate, expand their domains to the anterior (Welsh et al., 2007). These results suggest antagonism of Fgfs by *Spry2* affects a number of networks in the palate leading to gross changes in their expression patterns. Further analysis will be required to determine, which other factors are involved with the high rate of cleft palate in the piebald deletion mice. Although Fgf signaling is necessary for palate development, its action appears to require fine-tuning by repressors for normal palatogenesis to occur.

## Shox2 network

*Fgf10* expression is down-stream of the homeobox transcription factor *Shox2* (Yu et al., 2005). The *Shox2* gene is expressed exclusively in the anterior mesenchyme region of the developing secondary palate, with its highest expression occurring during the early stages of palate development. Mice deficient in the *Shox2* gene exhibit a rare form of cleft palate where the cleft only occurs in the anterior part of the secondary palate. Expression of a number of genes critical for palatogenesis such as *Jag2, Lhx8, Osr2, Pax9, Tgfb3*, and *Msx1* and its down-stream target *Bmp4* do not change in the *Shox2*^−/−^ palatal shelves (Yu et al., 2005). However, expression domains of both *Fgf10* and *Fgfr2c* are altered, which corresponds with altered proliferation and apoptosis within the anterior palate in the *Shox2* null mice (Yu et al., 2005). These data indicate altering the expression of genes only in one area of the palate can lead to clefting only in that specific area. However, *Msx1* is also expressed in the very anterior region of the palatal shelves and yet in *Msx1* null mice a complete cleft of the secondary palate is observed (Zhang et al., 2002) as it is in the *Fgf10* ^−/−^ mice (Rice et al., 2004; Alappat et al., 2005). It is not known precisely why *Shox2* exhibits this unusual anterior cleft. It may be that increased *Fgf10* expression is sending a signal to the posterior palate to fuse (Yu et al., 2005).

While regulation of the *Shox2* gene has been the subject of recent investigations in palate development, a complete understanding of *Shox2* regulation remains elusive (Yu et al., 2005). Blocking of Bmp signaling with the antagonist Noggin results in a down-regulation of *Shox2* expression within the exposed anterior mesenchyme at E12.5. Exposure of palatal mesenchyme to Bmp4, Bmp2, and Shh in culture is not sufficient to induce *Shox2* expression. These data suggest Bmp signaling is not capable of inducing *Shox2* expression on its own but is necessary for normal *Shox2* gene expression (Yu et al., 2005).

## Ephrin signaling

Ephrin-B1 belongs to the transmembrane B-type subfamily of Eph/Ephrin signaling molecules (Davy and Soriano, 2005). These signaling molecules have the ability to carry out bidirectional signaling. Hence, cells expressing the Eph receptor tyrosine kinase can receive a forward signal whereas cells expressing ephrin (Efn) can be transduced to receive the reverse signal (Bush and Jiang, 2012). The *Efnb1* gene is expressed in the mesenchyme of the anterior palate throughout the secondary palate development. *Efnb1* forward signals to regulate anterior palatal shelf outgrowth by promoting cell proliferation through the activation of ERK/MAP pathway (Bush and Soriano, 2010). Both *Efnb1* null mice and *Efnb1^+/−^* heterozygous female mice develop cleft palate with decreased cell proliferation in the anterior palatal mesenchyme (Bush and Soriano, 2010). The *Efnb1* gene is X-linked and the *Efnb1^+/−^* heterozygous embryos exhibit mosaic pattern of *Efnb1* expression in the palate that correlates with a mosaic pattern of proliferation and hence a more severe dysmorphogenesis of the palatal shelves compared to *Efnb*^*−/−*^ null mice. *Efnb1* is primarily expressed in the anterior mesenchyme, although the cleft palate phenotype was along the entire axis (Bush and Soriano, 2010). In contrast, loss of *Shox2* (described above) which is also anteriorly restricted in the palate, induces a unique anterior-delimited cleft palate (Yu et al., 2005). Hence, reduced *Efnb1* signaling in the anterior palatal mesenchyme in *Efnb*^*−/−*^ null or *Efnb1^+/−^* heterozygous embryos may cross a threshold A–P position at which initiation of fusion is required (Bush and Soriano, 2010).

## Tgf-β pathway

The role of *Tgf*-β family members in palatal shelf growth and fusion is an area that has been well-studied. Loss ofxd function of *Tgf-β2* (Sanford et al., 1997), *Tgf-β3* (Kaartinen et al., 1995; Proetzel et al., 1995; Martínez-Álvarez et al., 2000b), and *Tgf-β* receptors *Tgfbr1* (Dudas et al., 2006), *Tgfbr2* (Ito et al., 2003; Xu et al., 2006) are known to cause cleft palate. More detailed information on this pathway was reviewed recently in Bush and Jiang (2012). In the context of this review, we will only highlight the anterior and posterior-specific roles of these pathway members. The *Tgf* type I receptor *Alk5* is expressed exclusively in the anterior palatal epithelium and its activation in *Tgf*-*β3^−/−^* palatal epithelium rescues palatal fusion, whereas loss of *Alk5* function in epithelium of wild-type palatal shelves prevents palatal fusion (Dudas et al., 2004). Interestingly, fusion of the posterior parts of palates is predominant following activation of *Alk5* at E14 whereas its activation at E13.5 also facilitates fusion in the anterior region (Dudas et al., 2004). Thus, there appears to be an anterior to posterior direction of palatal fusion (Taya et al., 1999) with *Tgf*-*β3* signaling mediated by Alk5 in the anterior epithelium (Dudas et al., 2004). The homozygous knock-in of *Tgf-β1* in the *Tgf-β3* locus partially rescues the cleft palate phenotype of *Tgf-β3^−/−^* mice in the anterior palate (Yang and Kaartinen, 2007). Since *Tgf-β1* is expressed in the palatal epithelium along the A–P axis (Yang and Kaartinen, 2007), its partial rescue of anterior palatal fusion may also be mediated via Alk5 signaling in *Tgf-β3^−/−^* mice. Recent findings show that craniofacial abnormalities in *Tgfbr2* ^−/−^ mice is prevented following genetic manipulation of an alternative non-canonical *TGF*-β signaling pathway through Alk5/*Tgf* type III receptor complex and SMAD-independent TRAF6/TAK1/p38 signaling (Iwata et al., 2012b). The role of *Tgf-β3* in apoptosis of medial edge epithelium (MEE) is well-established (Martínez-Álvarez et al., 2000a,b) and *Tgf-β3* synthesized at the MEE facilitates accumulation of chondroitin sulfate proteoglycans at apical surface of MEE (Gato et al., 2002). Loss of *Tgf-β3* disrupts the normal distribution of E-cadherin, α-, and β-catenins in MEE and impairs cell-cell adhesion (Tudela et al., 2002). Conditional deletion of β-catenin in the epithelium in K14-Cre transgenic mice suppresses canonical Wnt signaling giving rise to an abnormal and persistent MES. Loss of β-catenin also induces down-regulation of *Tgf-β3* and inhibition of apoptosis in MEE that subsequently leads to a cleft palate phenotype (He et al., 2011). Hence, region specific expression of *Tgf-β3* is essential for proper palate development and these findings highlight the interplay between the various pathways that govern palate development.

## Posterior-specific gene expression

**Figure 3:**
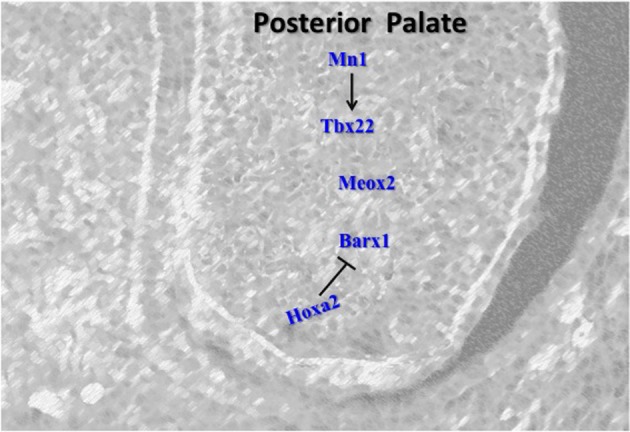
**Schematic representation of the key regulators in the posterior palate.** Barx1 and Tbx22 induce cell proliferation in the posterior palate. Hoxa2 also controls the expression of Barx1 in the early stage of palatogenesis. Mn1 positively regulates Tbx22 and represents the first network determined to specifically regulate the level of proliferation in the posterior palate. Meox2 plays role in fusion of the posterior palate.

Although many genes important in palate development have regional specific expression and are expressed predominantly in the anterior palate, several genes are also important in the posterior region (Figure 3).

## Meox2 network

*Meox2* is a homeobox transcription factor with a posterior-specific expression pattern that becomes increasingly localized to the extreme posterior region of the palate as development proceeds (Jin and Ding, 2006; Li and Ding, 2007). Mice lacking the *Meox2* gene exhibit a low penetrance of cleft palate that results from a novel mechanism. Palate shelves grow, elevate and fuse; however, fusion is weak and as the craniofacial region expands the palate shelves pull apart from one another leading to a cleft palate specifically in the posterior region. Histological analysis of the cleft palates clearly show the palate shelves were completely absent of the medial epithelial edge and were composed solely of mesenchyme. The mechanism responsible for the post-fusion cleft palate is not completely clear, but may involve a palatal growth defect in the posterior palate that prevents the palates from being able to keep up with the rest of craniofacial development (Jin and Ding, 2006). Alternatively, the loss of *Meox2* may lead to improper palatal fusion and a weak seam that does not stand up to the mechanical forces of craniofacial development (Jin and Ding, 2006). *Meox2* has been reported to be *Tgf-β* responsive in the mammary epithelial cells by inhibiting epithelial cell proliferation by binding to the promoter of p21 through *Tgf-β*/Smad signaling pathway (Valcourt et al., 2007). Interestingly, Xu et al. (2008) showed that p21 is required for Smad4 mediated p38 MAPK pathway for apoptosis and MES degeneration. These works suggest that *Meox2* could play a role in *Tgf-β3* mediated fusion. However, these mechanisms need to be investigated further.

## Tbx22 network

*Tbx22* also has a posterior-specific expression profile within the developing palate (Herr et al., 2003). The *Tbx22* gene is a T-box protein that acts as a transcription factor regulating the expression of down-stream genes. Alterations in the *Tbx22* gene are a common single cause of cleft palate in humans (Marçano et al., 2004). A missense mutation in the *Tbx22* gene is responsible for X-linked cleft palate (Marçano et al., 2004), whereby the missense mutation affects the ability of Tbx22 to bind DNA and subsequently act as a transcriptional repressor (Andreou et al., 2007). This mutation is believed to prevent the SUMO-1 enzyme from sumoylating the Tbx22 protein. In the absence of this post-translational modification, Tbx22 has a much lower affinity for its DNA binding sequence (Andreou et al., 2007), cannot recognize the DNA sequence and bind appropriately, and does not perform its normal repressor functions. Notably, SUMO-1 is again implicated in regulating palatogenesis. Based on the number of important genes known to require sumoylation to function properly, haploinsufficiency of SUMO-1 is not surprisingly linked to cleft palate phenotype (Alkuraya et al., 2006).

*Tbx22* expression in *Spry2* piebald mutants is affected as discussed above. In the absence of this Fgf antagonist, the expression of *Tbx22* fails to expand from the most posterior regions of the palate at E14.5. This altered expression profile coincides with a posterior shift in the expression of *Msx*1 as well as an increase in proliferation throughout the palate shelves (Welsh et al., 2007). The 5′ regulatory region of the *Tbx22* gene contains putative *Msx1* binding sites (Herr et al., 2003), however, *Msx1* null mice do not show an expansion of the *Tbx22* expression domain (Fuchs et al., 2010), and *Tbx22* null mice are not reported to have increased *Msx1* expression (Pauws et al., 2009). Palatal *Tbx22* expression has been demonstrated to be independent of Fgf signaling, but was reported to be repressed in culture by exogenous *Bmp4* (Fuchs et al., 2010). Taken together this suggests a system whereby *Msx1* is involved in regulating *Bmp4* expression which subsequently plays a role in the repression of Tbx22 expression, leading to a posterior-specific expression pattern.

Liu et al. describe a novel molecular network involving the *Tbx22* (Liu et al., 2008). The transcription factor Mn1 has a medial-posterior-specific expression profile that generally overlaps the *Tbx22* expression profile. Loss of one or more copies of *Mn1* leads to craniofacial abnormalities including a cleft secondary palate. In the *Mn1* null embryos, *Tbx22* expression decreases in the posterior region of the palate, and Mn1 directly regulates the expression of *Tbx22* in the palate (Liu et al., 2008). A marked decrease in proliferation in the medial and posterior palate shelves also occurs, and is believed to be due in part to the regulation of a separate gene target (*Ccnd2*) by Mn1 (Liu et al., 2008). This represents the first network determined to specifically regulate the level of proliferation in the posterior palate. Tbx22 expression appears to be regulated by at least two factors; *Msx*1 acts as a repressor, while Mn1 acts as an activator, and together they determine the specific expression domain of Tbx22 in the posterior region of the palate.

## Barx1 network

*Barx1* expression has a predominantly posterior expression profile that is complementary to the anterior expression of *Msx1* (Barlow et al., 1999; Welsh and O'Brien, 2009). This region-specific expression is initially set up in the branchial arches where *Msx1* expression is localized to the distal regions of the first brachial arch and *Barx1* expression is localized proximally (Barlow et al., 1999). The A–P axis derived from the regional expression of *Msx1* and *Barx1* is believed to result from the relative strength of Bmp and Fgf signaling (Welsh et al., 2007). The expression of *Barx1* is altered in a number of knockout mouse lines. Loss of *Spry2* via the piebald deletion not only affects *Msx*1 and *Tbx22* expression, but also leads to an anterior expansion of *Barx1* expression that may be involved in the increased cell proliferation seen in these palates (Welsh et al., 2007). The loss of the Bmp receptor *Bmpr1a* also leads to an expansion of the region in the palate expressing *Barx1* (Liu et al., 2005). *Hoxa2* null embryos have increased *Barx1* expression at the early stages of palate development. An increase in the level of cell proliferation in the posterior region of the palate is also observed in *Hoxa2* null mice (Smith et al., 2009). The alterations in both Fgf and Bmp signaling causing altered *Barx*1 expression support the view that regulation of the regional expression of *Barx1* involves both families of signaling molecules. Evidence for this comes from *Tp63^−/−^* mice where expression of *Fgf8* at E11.5 in the anterior region of maxillary processes is down-regulated, which coincides with a reduced anterior expression of its target gene *Barx1* (Thomason et al., 2008). In contrast, the *Tp63^−/−^* mice (which exhibit cleft lip and palate phenotype) show increased expression of *Bmp4* in the anterior region of the maxillary processes at E10.5 and E11.5. *Barx1* is also regulated by relative levels of *Fgf8* and *Bmp4* in developing chick facial primordia where BMPs reduce *Barx1* expression and antagonize Fgf-8 signaling (Barlow et al., 1999).

### At what stage does the antero-posterior molecular signaling get established?

An intriguing question during palatal development is when does the antero-posterior molecular signaling get established? Although answer to this remains elusive, available data indicates a much earlier time in development and prior to palatal shelf formation. The anterior localization of *Msx1* and posterior localization of *Barx1* is set to be determined in the first branchial arch where *Msx1* is localized to distal and *Barx1* to the proximal region (Barlow et al., 1999). In early mice palatal development, *Barx1* expression is visible in the posterior region extending through almost three quarters of the developing palatal shelves, whereas *Msx1* is restricted to a narrow anterior region of the developing palate (Welsh and O'Brien, 2009). Following rostral expansion, the anterior palate extends with the expression of *Msx1* and the first molar tooth bud serves as the posterior boundary of this extended anterior expression (Welsh and O'Brien, 2009). Hence, genes expressed in the presumptive hard and soft palate appear to be set up earlier along an A–P axis in the branchial arches. Consistent with this expression along an A–P axis in the first arch, *Msx1* plays a role in incisor development and *Barx1* in molar tooth development (reviewed in Mitsiadis and Smith, 2006). Interestingly, Bmp-Fgf signaling also governs tooth development in a gradient manner along an A–P axis (Mitsiadis and Smith, 2006). It is likely these genes play a similar role in the orofacial structures. Indeed similar to its role in palate development where *Bmp4* is required to prevent the premature apoptosis of palatal epithelium, *Bmp4* is essential in blocking apoptosis in the dental epithelium in a *Msx1*-dependent manner regulated by *Tgf-β* type I receptor Alk-5 (Zhao et al., 2008).

The transcription factor Tp63 regulates the expression of *Bmp4* in the anterior palate and loss of *Tp63* in the maxillary processes in the medial region from which the palatal shelves originate, results in improper Bmp signaling and a cleft palate phenotype (Thomason et al., 2008). However, conditional inactivation of *Bmp4* in the early maxillary mesenchyme using *Nestin cre;Bmp4* ^*null/flox*^*(n/f)* mice did not disrupt secondary palate development but resulted instead in isolated cleft lip (Liu et al., 2005). Loss of *Bmpr1a* in facial primodia of *Nestin cre;Bmpr1a n/f* embryos, which did not impact *Msx1* expression, resulted in reduced mesenchymal cell proliferation in maxillary process prior to the onset of secondary palate outgrowth resulting in smaller palatal shelves and subsequent cleft palate at birth (Liu et al., 2005). In contrast, tissue-specific loss of *Bmpr1a* in palatal mesenchyme in *Osr2-IresCre;Bmpr1a^f/f^* mutant mice results in reduced expression of *Msx1* and an up-regulation in *Bmp4* leading to submucous cleft of the hard palate (Baek et al., 2011). Thus, the BMP family highlights the complexity of signaling involved in their early tissue specific role in orofacial development. Further early fate determination studies are needed using lineage specific animal models to characterize the complex signaling during early palate development to clearly determine the origins of the A–P molecular signaling.

### Crosstalk between networks/pathways

Palatal elevation and fusion is governed by transcription factors, various growth factors and their receptors forming interconnected network of molecular pathways. Relative signals or gradients are strictly required to ensure proper development. The anterior and posterior palatal tissues being the future hard and soft palates differ in function and structure, show difference in the expression patterns along the A–P axis. Numerous pathways/networks in the palate clearly display reciprocal signaling between the epithelium and mesenchyme. Genes expressed exclusively in the epithelium have been reported to regulate cellular processes and gene expression in the mesenchyme, and vice versa, such that reciprocal signaling occurs between the epithelium and mesenchyme (Zhang et al., 2002; Yamamoto et al., 2003; Rice et al., 2004; Nawshad et al., 2007). In recent years it has become increasingly evident that gene expression in the developing palate not only displays epithelial-mesenchymal specificity, but also anterior–posterior (A–P) and oral-nasal specificity (reviewed in Murray and Schutte, 2004; Hilliard et al., 2005; Bush and Jiang, 2012). Interestingly, *Tp63* null mutants have abnormal outgrowth of the palate initially but by E12.5 the A–P specific expression patterns were normal despite abnormal shelf growth confirming the importance of setting up and maintaining the A–P axis (Thomason et al., 2008). In addition to their localized expression, genes have been reported to elicit different responses in different regions of the palate. For example, while exogenous *Fgf10* alters proliferation in the anterior palate, it has no effect on proliferation in the posterior region of the palate (Yu et al., 2005). Such localized expression and function phenomena clearly highlight the importance of regional patterning and differentiation within the palate at the molecular level. Overall, the number of genes involved in the development of the palate that display strictly regulated domains of expression is clear evidence of regional differentiation within the palate. *Msx1* and *Bmp4* function in an autoregulatory loop mechanism in the anterior palatal mesenchyme. *Bmp4* induces *Shh* expression in the epithelium which signals backs to mesenchyme to positively regulate Bmp2 to enhance cell proliferation in the mesenchyme (Zhang et al., 2002). Cross talk also exists between *Bmp7* and *Shh*, which plays a role in refining the expression domain of both genes (Han et al., 2009). *Tbx3* and *Bmp4* regulate each other's expression in the palate. *Tbx*3 inhibits the expression of *Bmp4* while *Bmp4* induces *Tbx*3 expression and regulates the levels of cell proliferation in the anterior palatal mesenchyme (Lee et al., 2007). Regulatory feedback loop exists between *Fgfr1b* and *Wnt11*. *Fgfr1b* represses expression of *Wnt11* whereas *Wnt11* signaling molecule negatively regulates *Fgfr1b* expression (Lee et al., 2008). Balance is titled toward or away from *Fgfr1b*, to respectively allow cell proliferation to proceed (at E13.5) or to recede (at E14) and fusion to occur (Lee et al., 2008). Unlike the anterior palate, molecular mechanisms of palatal outgrowth in the posterior palatal regions are not yet well established. Mn1 directly regulates the expression of *Tbx22* in the palate (Liu et al., 2008) and *Msx1* acts as a repressor of *Tbx22* in the palate (Welsh et al., 2007) which together determines the posterior expression domain of Tbx22 in the palate.

## Anterior palate-signaling center

**Figure 4:**
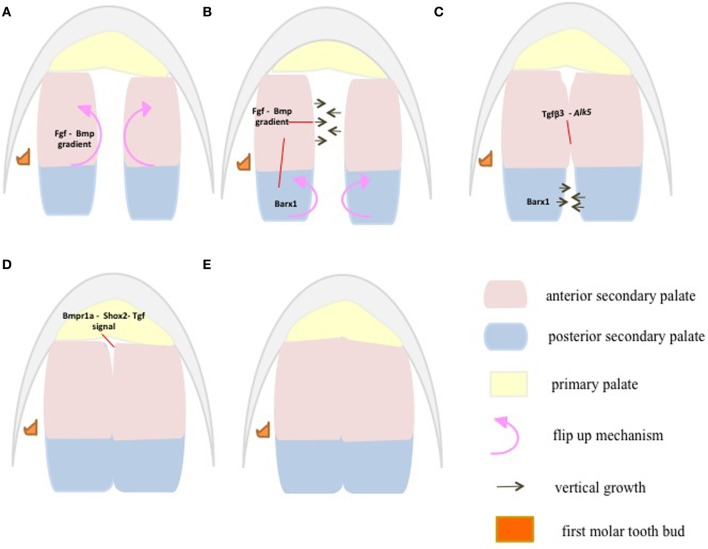
At E13.5, the anterior palatal shelves first flip up to orient vertically when the posterior palatal shelves are still lying horizontal to each other **(A)**. At E14, the posterior palatal shelves follow the anterior palatal shelves in orienting vertically, whereas the anterior palates begin to grow vertically toward each other to make contact **(B)**. At E15, the anterior palatal shelves have made contact and fused, whereas the posterior shelves grow vertically **(C)**. At E15.5, both the anterior and posterior palates have fused **(D)**. At E16, the fusion between the primary and secondary palate occurs at the future secondary choana **(E)**. Palatal shelves are divided into anterior (pink) *Msx1* and posterior (aqua) *Barx1* expression domains **(A–E)** representing future hard and soft palate, respectively. Fgf-Bmp gradients/thresholds maintain proper palatal growth and fusion through proliferation. Anterior *Fgf10* and Bmps control proliferation via *Shh* expression. This directs anterior palate flip up and vertical growth at E13.5–E14 **(A,B)**. Anterior Fgf-Bmp gradients along with posterior *Fgf8* regulate proliferation and growth via *Barx1* in the posterior palate at E14–E15 **(B,C)**. Fusion is initiated at the anterior palate by *Tgf-β3* through its receptor **(C)**. Then the fusion extends posteriorly through *Tgf-β*-Meox2 **(D)**. The fusion between the primary and secondary palate marks the completion of palatal fusion at E16 **(D,E)**, via *Bmpr1a* mediated *Shox2* and *Tgf β* signaling through its receptors.

Critical events such as elevation, maturation and fusion of secondary palatal shelves follow an anterior to posterior sequence (Taya et al., 1999; Dudas et al., 2004) (Figure 4). During mouse palate development, at embryonic day E13.5–E14, the anterior palate orients horizontally above the tongue when the posterior palate is still lying vertically (Kaufman, 1992) providing a clear indication of the more dynamic growth in the anterior palate compared to the posterior palate. In addition, the initial site of apposition and subsequent fusion of the palatal shelves occur first in the anterior half of the palate and the sequence of palatal closure may be result of signaling activity along the A–P axis.

Although *Shox2* expression remains anterior-specific throughout palatogenesis, it displays a dynamic pattern of expression. At the initial stages of palate growth, *Shox*2 expression is only detected in the most extreme anterior regions of the palate (less than 25% of the length of the palate) (Yu et al., 2005; Li and Ding, 2007). As the palate shelves continue to grow, the expression of *Shox*2 expands until E14.5 when it covers the entire anterior palate and most of the medial palate (60% of the length of the palate shelf). Concurrent with the expansion of *Shox2* expression, the region of the palate expressing the posterior-specific gene *Meox2* shrinks (Li and Ding, 2007). This phenomenon demonstrates normal development of the anterior palate requires recruitment of cells from the posterior, which are converted into *Shox2* anterior-specific cells. This has been suggested to be due to a repression of *Meox2* by *Shox2* or a down-stream target of the *Shox2* pathway (Li and Ding, 2007).

The rugae have been suggested to play an important role in organizing and maintaining the A–P axis (Welsh et al., 2007; Pantalacci et al., 2008; Welsh and O'Brien, 2009). Rugae have been shown to form in the region between the last formed and rugae 8 (the first rugae to form) in a sequential order (Pantalacci et al., 2008; Welsh and O'Brien, 2009). Rugae 8 has been denoted the “boundary rugae” as it appears to act to separate the anterior and posterior domains of the palate. Throughout palatal development, expression of the anterior specific markers *Shox2* and *Msx1* remain anterior to rugae 8 and *Meox2* and *Tbx22* stay posterior of this boundary (Pantalacci et al., 2008; Welsh and O'Brien, 2009). This rapid expansion of the palate anterior to rugae 8 provides an alternate explanation for the anterior growth of the palate to the one detailed above by Li and Ding (2007). The major difference is that Li and Ding did not detect differences in the proliferation rates of the anterior and posterior palate, while Pantalacci et al. (2008) did detect a higher level in the anterior palate. Which theory is deemed to be correct will require further investigations.

Mice lacking expression of either *Wnt5a* or its noncanonical receptor Ror2 were found to exhibit a cleft palate (Schwabe et al., 2004; He et al., 2008). In addition, both genes were shown to exhibit an expression pattern whereby their expression was higher in the anterior palate than the posterior palate (He et al., 2008), with *Wnt5a* detected exclusively in the mesenchyme (Paiva et al., 2010). The *Wnt5a* signal was consequently shown to act as a chemoattractant causing cells to migrate from the posterior region of the palate toward the anterior region. Evidence suggests that Ror2 mediates the role of *Wnt5a* in the palatogenesis (He et al., 2008). As discussed above, *Wnt5a* also regulates the expression of *Bmp4* and *Shh*, both of which play important roles in the development and growth of the anterior palate (He et al., 2008). Hence, simple upregulation of *Shox2* and downregulation of *Meox2* may not result in the conversion of posterior cells to anterior cells if the cells are migrating toward the higher *Wnt5a* signal. As cells enter the anterior region of the palate, *Wnt5a* and potentially other factors may act to alter the expression profile of genes in the cells, causing them to transdifferentiate into anterior-specific palate cells.

Collectively, these recent discoveries, suggest that cells may migrate—first from the posterior region of the palate to the anterior region of the palate and then to the oral region of the anterior palate—underscore the dynamic processes taking place during palate development. While at any given time cells display a specific set of genes that determine how they react to external stimuli, this set of factors continually changes as development proceeds. In addition, the migration to the anterior region of the palate specifically lends further support to the theory the anterior palate plays a role as a signaling center acting to regulate palatogenesis as a whole. It also demonstrates the importance of maintaining a proper anterior to posterior axis in the palate development.

## Conclusion

Regulation of palate development appears to be the result of distinct pathways in the anterior and posterior regions of the palate. Development and maintenance of expression of these regional-specific genes is crucial to normal palate development. Anterior- and posterior- specific genes appear to act in a mutually exclusive manner by either directly or indirectly inhibiting reciprocal expression.

Recent findings show posterior palate cells maintain the ability to transform into anterior specific cells upon migration. These data demonstrate the plasticity of these cell populations despite their differential responses to external stimuli.

To date, researchers have often limited their investigations to determining levels of gene expression of putative targets. However, the future of palate research will need to consider the regional specificity of target genes. An important focus of new studies should be to examine the expression domains of potential targets along the A–P axis, as expansion or limitations of these domains can dramatically affect palate development.

### Conflict of interest statement

The authors declare that the research was conducted in the absence of any commercial or financial relationships that could be construed as a potential conflict of interest.
